# Alterations in Spatiotemporal Parameters in Patients With Lower‐Limb Amputation: A Systematic Review With Meta‐Analysis

**DOI:** 10.1002/pri.70327

**Published:** 2026-08-21

**Authors:** Luis Augusto Mendes Fontes, Guilherme Auler Brodt, Adna Thalita do Nascimento Silva, Isabela Regina de Lima Andrade, Livia Barboza de Andrade

**Affiliations:** ^1^ Postgraduate Program in Integrative Health Instituto de Medicina Integral Professor Fernando Figueira (IMIP) Recife Pernambuco Brazil; ^2^ Biomechanics University of Caxias do Sul Caxias do Sul Brazil; ^3^ Specialized Rehabilitation Center Instituto de Medicina Integral Professor Fernando Figueira (IMIP) Recife Pernambuco Brazil

**Keywords:** amputees, balance, cadence, gait, gait analysis

## Abstract

**Objectives:**

To evaluate differences in spatiotemporal gait parameters in individuals with transfemoral (TFA) and transtibial amputation (TTA) compared with physically able individuals.

**Methods:**

This systematic review with meta‐analysis was conducted according to the MOOSE guidelines. Cross‐sectional studies or clinical trials that assessed spatiotemporal gait parameters in adults with unilateral TFA or TTA were included. Searches were performed in Medline (via PubMed), CINAHL, Scopus, LILACS, Cochrane Library, and Embase using descriptors related to amputation and gait. Risk of bias was assessed using the Joanna Briggs Institute scale for cross‐sectional studies, whereas the meta‐analysis was performed using quantitative data for the following outcomes: walking speed, step length, stride length, step width, cadence, stance time, swing time, step time, or stride time.

**Results:**

A total of 12 cross‐sectional studies involving 150 individuals with amputation (86 TTA and 64 TFA) and 138 healthy controls were included. Meta‐analysis demonstrated a significant reduction in walking speed (mean difference of −0.24; 95% CI −0.32 to −0.17; *p* < 0.0001; *I* ^2^ = 61%) and cadence (mean difference of −6.01; 95% CI −9.69 to −2.34; *p* = 0.001; *I* ^2^: 54%) in patients with amputation compared with healthy individuals. A reduction in stride length (mean difference of −11.71; 95% CI −23.37 to −0.04; *p* = 0.05; *I* ^2^: 85%) and an increase in step width (mean difference of 5.22; 95% CI 2.99 to 7.45; *p* < 0.0001; *I* ^2^: 71%) were also observed. Step time showed no significant difference between groups (mean difference of 0.06; 95% CI −0.01 to 0.14; *p* = 0.11; *I* ^2^: 93%). Patients with TFA amputation exhibited greater impairment in gait variables, particularly cadence, when compared with a healthy individual.

**Conclusions:**

Patients with lower limb amputation present with functionally compromised gait, characterized by reduced walking speed. Increased step width and reduced stride length are findings that may suggest compensatory strategies during gait and improved balance, which are important requirements for amputee patients. These findings reinforce the need for rehabilitation interventions focused on improving propulsion and postural safety.

**Trial Registration:**

PROSPERO: CRD42024620098

## 1 Introduction

Lower‐limb amputation is a transformative event that imposes substantial biomechanical and functional challenges that affect postural control and locomotor efficiency. Gait analysis in individuals with amputation is essential for quantifying these alterations, which are critical in transtibial (TTA) and transfemoral amputations (TFA). In these patients, loss of joint and muscular function results in kinetic and spatial asymmetries, with direct effects on stability and load distribution (Seth et al. 2021).

Individuals with amputation develop a complex set of gait strategies, including changes in step length, walking speed, and increased double‐support time on the intact limb, to maintain dynamic balance and forward progression and compensate for the limited propulsive capacity of the prosthesis (Hak et al. 2013; Mira et al. 2025). The level of amputation dictates the magnitude of the challenge, with more proximal amputations (e.g., TFA) often resulting in greater asymmetry and higher metabolic demand due to the absence of the knee joint and consequent difficulties in controlling the center of mass (Faizan and Pal 2023).

Despite recognition of these adaptations, a consistent and comparative quantification of gait parameters across different amputation levels and physically able individuals remains challenging in clinical practice and research. Substantial methodological variability, including differences in assessment protocols and heterogeneity of the studied populations, limits the consolidation of robust evidence on effective interventions (e.g., propulsive strength training or the use of advanced devices) for improving gait symmetry and safety (Seth et al. 2021).

Unlike past systematic reviews on the subject, the present study is necessary because there is a gap in the literature to clearly understand the magnitude of the difference in gait parameters between patients with different levels of lower limb amputation and physically able individuals, in order to summarize such scientific evidence.

## 2 Methods

### 2.1 Protocol and Registration

This systematic review was conducted according to the guidelines for systematic reviews and meta‐analyses of observational studies (MOOSE) (Stroup et al. 2000). The protocol was registered in PROSPERO (CRD42024620098).

### 2.2 Eligibility Criteria

Studies were eligible when they: (1) employed a cross‐sectional design or reported baseline values from clinical trials; (2) assessed spatiotemporal or kinematic gait parameters during overground or treadmill walking in adults with unilateral transtibial (TTA) or transfemoral amputation (TFA); (3) included a comparison group of physically able individuals; and (4) provided numerical data for at least one of the following outcomes: walking speed, step length, stride length, step width, cadence, stance time, swing time, step time, or stride time. Comparisons of prosthetic components would not be included. Studies involving bilateral or congenital amputations, pediatric samples, or lacking quantitative gait measures were excluded.

### 2.3 Outcome Measures

Primary outcomes were spatiotemporal gait variables commonly used to characterize locomotor performance: walking speed, step length, stride length, step width, cadence, stance time, swing time, step time, or stride time. Data extraction was performed in relation to the prosthetic limb, since the objective of the study was to understand the difference in the behavior of the spatiotemporal gait variables of amputee patients, therefore with their prosthetic limb, when compared to healthy individuals.

### 2.4 Search Strategy

Comprehensive search strategies were constructed for each database using a combination of Medical Subject Headings (MeSH) and free‐text terms related to (1) lower‐limb amputation and (2) gait analysis. Synonyms within each concept block were combined using the Boolean operator “OR,” and the main concepts were joined using “AND”. For PubMed/MEDLINE, the complete, reproducible search string incorporated MeSH terms and title/abstract keywords as follows: (“Amputees”[Mesh] OR “Amputation”[Mesh] OR “amputee*” OR “lower limb amputation*” OR “lower extremity amputation*” OR “transfemoral” OR “transtibial” OR “above‐knee amputation*” OR “below‐knee amputation*” AND (“Gait”[Mesh] OR “Gait Analysis”[Mesh] OR “Walking Speed”[Mesh] OR “gait” OR “gait analysis” OR “walking speed”)).

Initially, the titles and abstracts were screened, and if necessary, the methods section was reviewed to determine eligibility. Retrieved articles were recorded in Mendeley, and duplicate records were identified and removed.

### 2.5 Study Selection

Searches were conducted independently by two researchers (L.M. and A.T.) across Medline (via PubMed), CINAHL, Scopus, LILACS, Cochrane Library, and Embase databases. Discrepancies were resolved through consensus. A third reviewer (L.B.) arbitrated unresolved disagreements.

### 2.6 Data Extraction

The following data were extracted using a standardized form: study identification (author, year), participant characteristics (age, sex, type of amputation level) and quantitative values for the predefined gait outcomes (walking speed, step length, stride length, step width, cadence, stance time, swing time, step time and stride time).

### 2.7 Risk of Bias

Risk of bias was assessed using the JBI Checklist for Analytical Cross‐Sectional Studies scale for cross‐sectional studies. The instrument assesses eight methodological domains related to sample selection, confounding, measurement procedures, and statistical reporting, with each item classified as low, high, or unclear risk (Munn et al. 2014). Risk of bias assessment was performed independently by two researchers; in cases of disagreement, a third researcher was consulted to resolve the issue.

### 2.8 Statistical Analysis

Meta‐analyses were performed using a random‐effects model, a choice justified by the anticipated clinical and methodological heterogeneity across gait analysis protocols and prosthetic components. Statistical heterogeneity was quantified using the *I* ^2^ statistic, where values of 25%, 50%, and 75% represented low, moderate, and high heterogeneity, respectively. To avoid unit‐of‐analysis errors in studies reporting data for both prosthetic and intact limbs separately, data were extracted exclusively from the prosthetic limb to ensure independence of observations.

## 3 Results

### 3.1 Study Description

**FIGURE 1:**
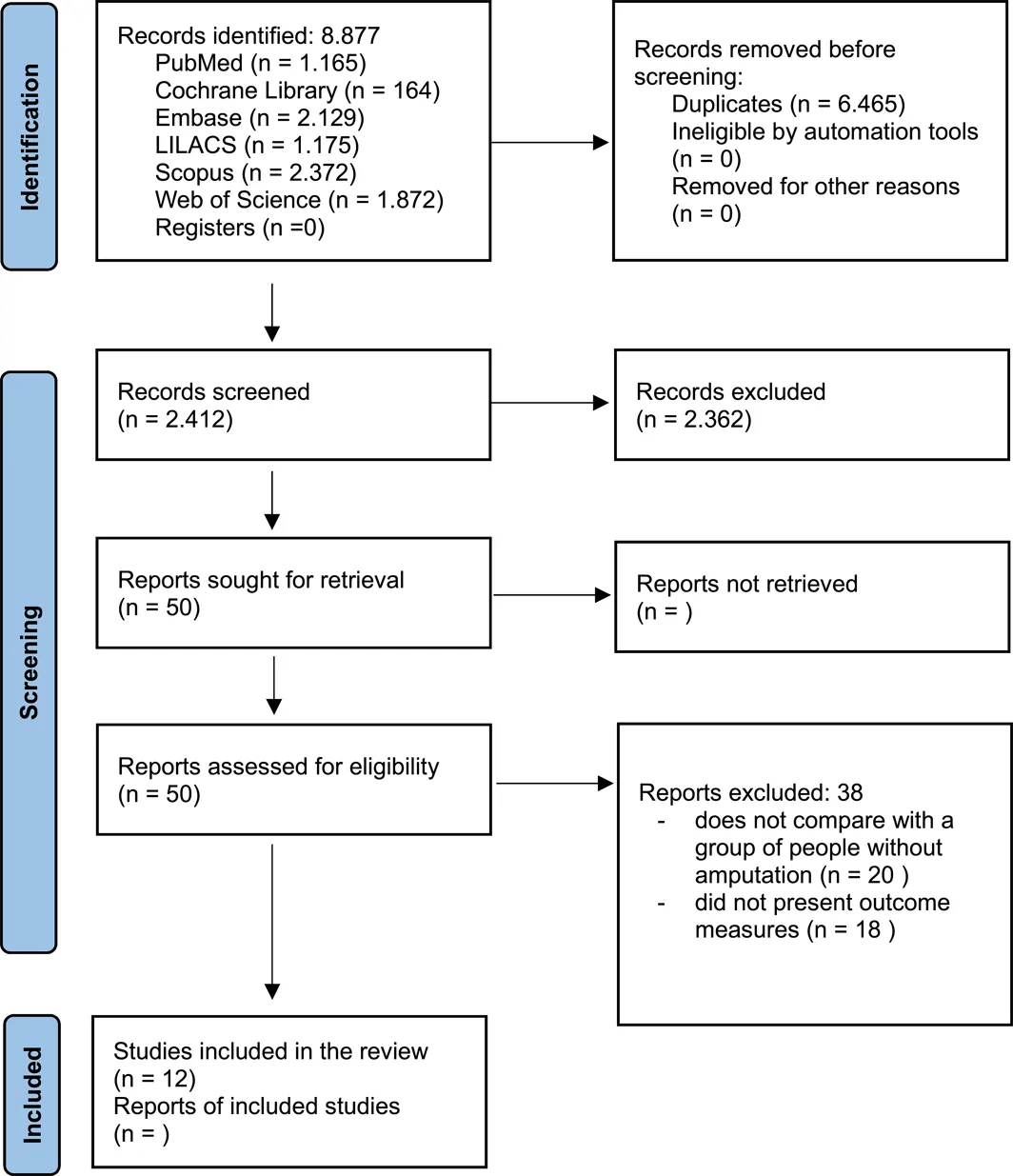
Flowchart of study selection.

A total of 8877 records were retrieved in the literature search. After duplicate removal and peer screening, 12 cross‐sectional studies were selected for inclusion in the systematic review (Figure 1). One hundred and fifty individuals with amputation—86 with TTA and 64 with TFA—with a mean age of 47.21 ± 14.83 years and 138 heath controls, were included, with a mean age of 40.93 ± 15.15. In the group of amputee patients, there were 76 men and 16 women in the studies that described the frequency of sex among patients. On the other hand, among healthy volunteers, there were 58 men and 20 women. All selected studies were carefully reviewed, and quantitative data were extracted for synthesis and meta‐analysis.

### 3.2 Methodological Quality Assessment

The risk of bias of the included studies demonstrated good methodological quality according to the JBI scale. It was analyzed by two researchers, and in case of disagreement, it was discussed with a third researcher to determine the risk of bias. Half of the 12 included studies presented a low risk of bias across all eight domains of the checklist. The risk of bias assessment of the 12 included studies revealed a predominantly high methodological quality, with most domains classified as low risk of bias. Half of the evaluated studies (*n* = 6; 50%) presented a low risk of bias across all established analytical criteria (Beyaert et al. 2008; Boonstra et al. 1993; De Pauw et al. 2020; Grumillier et al. 2008; Morgan et al. 2016).

Domain‐specific analysis demonstrated that criteria related to the clear definition of the sample, detailed description of subjects/setting, use of standard criteria for measurement of the condition, and appropriateness of statistical analysis achieved 100% low risk of bias across the entire sample. Conversely, the primary source of methodological heterogeneity was concentrated in domains associated with confounding factors. Confounding factor identification presented an unclear risk of bias in five studies (Nolan et al. 2003; Rao et al. 1998; Segal et al. 2011; Tokuno et al. 2003; Tura et al. 2010), while strategies to address these confounders were classified as unclear risk in four studies (Nolan et al. 2003; Rao et al. 1998; Segal et al. 2011; Tokuno et al. 2003).

High risk of bias was an infrequent finding in this review. High risk was recorded only for the domain regarding the validity and reliability of exposure measurement in the study by Nolan et al. (2003), and for the identification and management of confounding factors in the study by Tura et al. (2010). No other studies were classified as high risk for the remaining items assessed.

### 3.3 Demographic Characteristics of the Included Studies

All 12 included studies evaluated adults with TFA or TTA. The mean age of individuals with amputation was 46 ± 9.09 years, whereas the physically able bodies had a mean age of 44 ± 9.89 years. In most studies, sex distribution was clearly reported: 124 males (82.6%) and 26 females (17.4%) in the group with amputation, and 74.3% males and 25.7% females in the control group. The studies by (Beyaert et al. 2008; Boonstra et al. 1993) did not specify the sex distribution.

### 3.4 Clinical Characteristics of the Included Studies

#### 3.4.1 Spatial Parameters

Stride length was the most frequently reported variable: two studies involved TTA (mean of 121.25 ± 20.15 cm) and six involved TFA (mean of 140.5 ± 12.61 cm). Step length was reported in only one study with TTA (mean of 74 ± 9 cm), whereas step width was assessed in three studies involving TFA (mean of 18.8 ± 2.97 cm) and in one involving TTA (mean of 13.3 ± 2.9 cm).

#### 3.4.2 Temporal Parameters

Cadence, stance time, swing time, step time, and stride time were evaluated. Cadence was the most frequently assessed temporal outcome, with three studies reporting data on TTA (mean of 107.26 ± 3 steps per minute) and two on TFA (mean of 103.55 ± 0.63 steps per minute). Each of the remaining temporal variables was reported in only one study, except for step time in TTA, which was reported in two studies (mean of 0.66 ± 0.12 seconds).

#### 3.4.3 Walking Speed

Self‐selected gait speed was considered a composite variable because it reflects both spatial and temporal parameters. This was the most frequently assessed outcome at both amputation levels: four studies reported a mean gait speed of 1.11 ± 0.05 m s^−1^ for individuals with transfemoral amputation (TFA), while five studies reported 1.33 ± 0.13 m s^−1^ for transtibial amputation (TTA).

#### 3.4.4 Meta‐Analysis

Detailed meta‐analytic findings for each variable are presented below and illustrated graphically in the forest plots. Notably, formal subgroup comparisons revealed no statistically significant differences between individuals with transfemoral amputation (TFA) and those with transtibial amputation (TTA) for any evaluated outcome.

#### 3.4.5 Walking Speed

**FIGURE 2:**
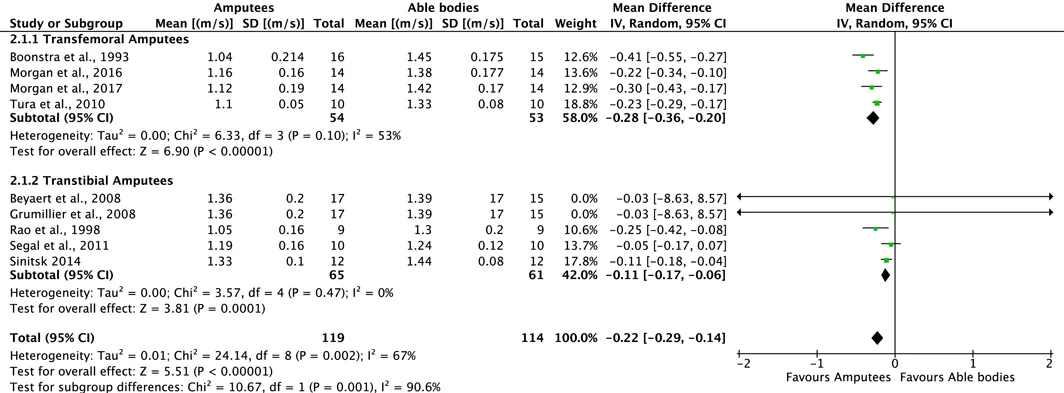
Forest plot of walking speed (m s^−1^). Forest plot for walking speed with the random‐effects model split into subgroups (TTA and TFA) and the overall effect. Squares represent the mean differences between the groups of each study; horizontal lines represent the 95% CIs; and the black diamond represents the pooled effect for each subgroup and for the overall effect. *I* ^2^ = 61%.

Nine studies investigating walking speed in individuals with TTA and TFA were included in the meta‐analysis. The pooled mean difference was −0.24 (95% CI −0.32 to −0.17; *p* < 0.0001; *I* ^2^: 61%), indicating a significant reduction in walking speed in the amputation group compared with controls (Figure 2).

#### 3.4.6 Cadence

**FIGURE 3:**
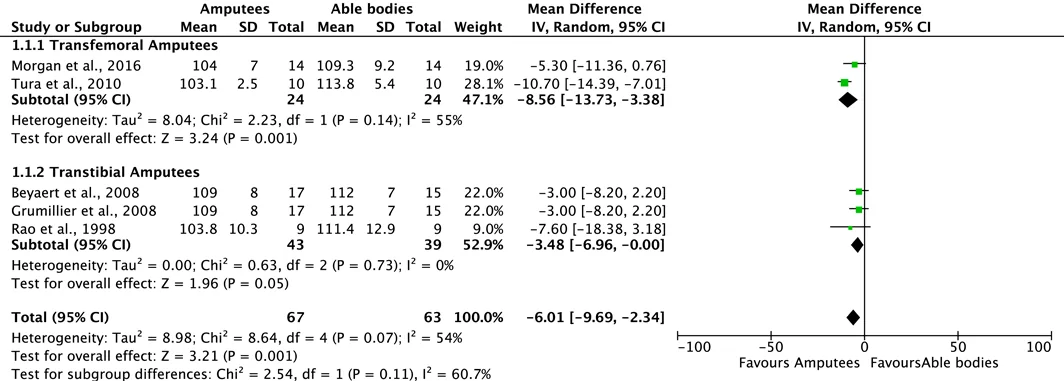
Forest plot of cadence (steps/min). Forest plot for cadence with the random‐effects model split into subgroups (TTA and TFA) and the overall effect. Squares represent the mean differences between the groups of each study; horizontal lines represent the 95% CIs; and the black diamond represents the pooled effect for each subgroup and for the overall effect. *I* ^2^ = 54%.

Five studies were included in the meta‐analysis of cadence. The pooled mean difference was −6.01 (95% CI −9.69 to −2.34; *p* = 0.001; *I* ^2^: 54%), demonstrating a significant reduction in cadence compared with the control group (Figure 3).

#### 3.4.7 Step Width

**FIGURE 4:**
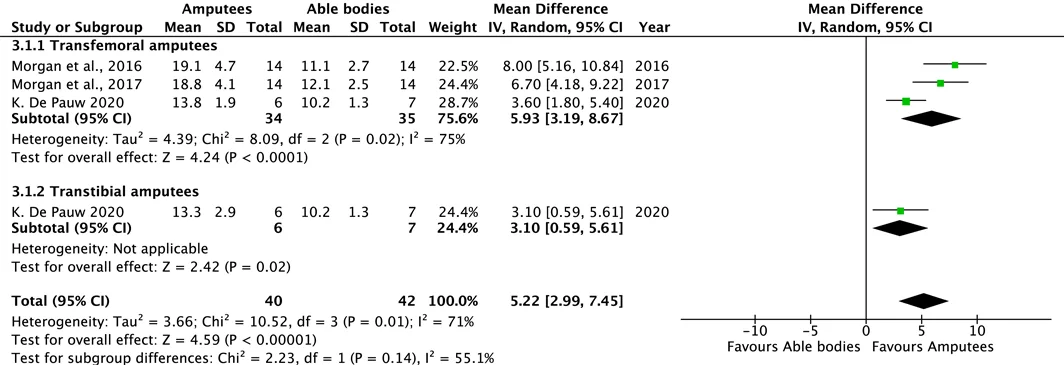
Forest plot of step width (cm). Forest plot for step width with the random‐effects model split into subgroups (TTA and TFA) and the overall effect. Squares represent the mean differences between the groups of each study; horizontal lines represent the 95% CIs; and the black diamond represents the pooled effect for each subgroup and for the overall effect. *I* ^2^ = 71%.

Four studies were included in the meta‐analysis of step width. The pooled mean difference was 5.22 (95% CI 2.99 to 7.45; *p* < 0.001; *I* ^2^: 71%), indicating a significant increase in step width compared with controls (Figure 4).

#### 3.4.8 Stride Length

**FIGURE 5:**
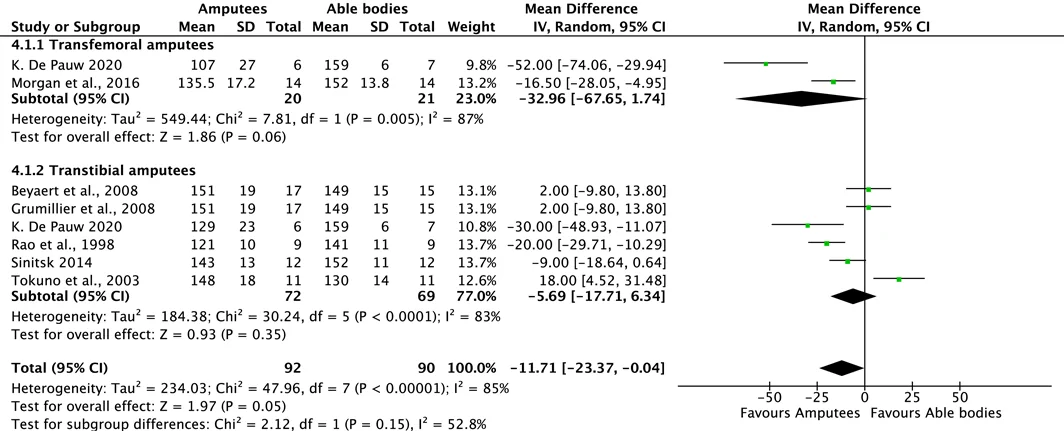
Forest plot of stride length (cm). Forest plot for stride length with the random‐effects model split into subgroups (TTA and TFA) and the overall effect. Squares represent the mean differences between the groups of each study; horizontal lines represent the 95% CIs; and the black diamond represents the pooled effect for each subgroup and for the overall effect. *I* ^2^ = 85%.

The meta‐analysis of stride length included eight studies. The pooled mean difference was −11.71 (95% CI −23.37 to −0.04; *p* = 0.05; *I* ^2^: 85%), indicating a significant reduction in stride length in the amputation group compared with the control group (Figure 5).

#### 3.4.9 Step Time

**FIGURE 6:**
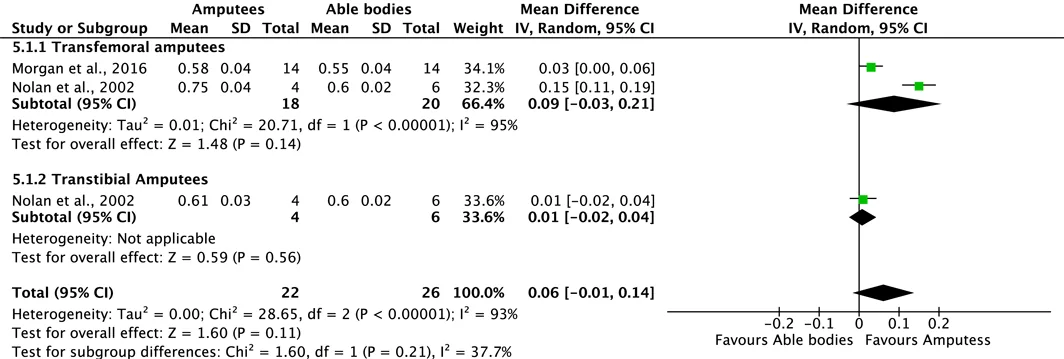
Forest plot of step time (s). Forest plot for step time with the random‐effects model split into subgroups (TTA and TFA) and the overall effect. Squares represent the mean differences between the groups of each study; horizontal lines represent the 95% CIs; and the black diamond represents the pooled effect for each subgroup and for the overall effect. *I* ^2^ = 93%.

Two studies investigating step time were included in the meta‐analysis. The pooled mean difference was 0.06 (95% CI −0.01 to 0.14; *p* = 0.11; *I* ^2^: 93%), indicating no significant difference between individuals with amputation and healthy controls (Figure 6). This finding should be analyzed carefully because of the small number of participants in both studies relative to the outcomes and their high heterogeneity.

## 4 Discussion

This systematic review synthesized evidence on spatiotemporal gait deviations in adults with unilateral lower‐limb amputation. Across the included studies, consistent alterations were observed in walking speed, cadence, stride length, and step width when compared with physically able individuals. Although heterogeneity and limited sample sizes constrain the precision of pooled estimates, the direction of effects across studies suggests a robust pattern of mechanical and temporal adaptations.

Consistent with previous findings (Buckley et al. 2013), our results demonstrated a lower mean walking speed in individuals with amputation than in controls. Walking speed is closely associated with mechanical power generated by the muscles that propel the body. In individuals with lower‐limb amputation, studies have also shown a reduction in plantarflexor moment (Soares et al. 2009). Moreover, individuals using prosthetic feet with hydraulic mechanisms may achieve higher walking speeds when compared to physically able individuals(De Asha et al. 2014; De Pauw et al. 2019), resulting in altered acceleration power during gait(Orekhov et al. 2019). However, this review did not systematically analyze prosthetic foot type, which may influence walking speed.

Decreased confidence in loading the prosthetic limb is another factor contributing to reduced walking speed and increased ground reaction force asymmetry. Previous studies have demonstrated a clear reduction in ground reaction force on the prosthetic side compared with physically able individuals, resulting in asymmetric weight‐bearing during gait (Silverman et al. 2008; Snyder et al. 1995). In addition, prosthetic components may influence gait performance as individuals using rigid ankle components exhibit reduced joint range of motion and consequent biomechanical disadvantage during the propulsive phase (Boonstra et al. 1993; De Pauw et al. 2020).

Reductions in walking speed are commonly accompanied by a decrease in cadence, given that the latter is speed‐dependent. Previous studies have demonstrated that lower‐limb amputees exhibit reduced cadence (Rowe et al. 2014). Furthermore, prior research indicated that approximately 60% of individuals with amputations report feeling insecure while walking and perceive a need to consciously focus on each step, suggesting greater gait vulnerability and a consequent reduction in step frequency (Miller et al. 2001), suggesting increased gait vulnerability and a consequent reduction in the number of steps per minute.

Spatial parameters, such as step length and width, also differed between individuals with amputation and controls. These alterations may be explained by changes in motor control, reduced sensory input, and impaired proprioception of the affected limb (Hordacre et al. 2015). Increased step width likely reflects an enlarged base of support, which is associated with maintaining dynamic balance during walking (Takada et al. 2025). In addition, reduced stride length may be associated with prolonged stance time, adaptive strategies to enhance anteroposterior stability, and diminished ankle propulsive mechanisms (Hak et al. 2014; Seth et al. 2021). These findings are consistent with previous studies demonstrating that gait in individuals with amputation is asymmetric (Uchytil et al. 2014) and may lead to secondary compensations, such as hip and knee osteoarthritis, on the intact limb due to chronic overload during walking (Gailey et al. 2008). However, we cannot infer a causal relationship between osteomyoarticular conditions because of the cross‐sectional design of our systematic review.

These findings must be interpreted with caution, primarily due to the limited sample sizes and the substantial statistical and clinical heterogeneity observed across the included studies. Such variability likely stems from the lack of standardization in patient profiles; even when stratified by amputation level (transfemoral vs. transtibial), participants may present with diverse baseline health conditions, further increasing variance. Moreover, the employment of disparate gait assessment methodologies across studies may have contributed to the inconsistent results. Although the type of prosthetic componentry was not restricted to avoid overly narrow inclusion criteria, this lack of control represents an additional factor that may have exacerbated the observed heterogeneity.

Furthermore, a significant limitation of this review is its exclusive focus on the anatomical level of amputation—transtibial versus transfemoral—without considering the specific types of prosthetic components used, such as mechanical versus microprocessor‐controlled knees or different carbon fiber foot designs. Since prosthetic technology directly modulates gait symmetry, energy return, and compensatory mechanisms, the absence of a more detailed analysis of these components prevents a more definitive conclusion about the actual impact of amputation on gait biomechanics. In addition, the review included cross‐sectional studies, thus losing the ability to infer causality. Furthermore, the cross‐sectional design of the included studies limits the inference of causality, and the high heterogeneity leads to limitations in the precision of the results. Additionally, the small number of studies relative to the time and step length makes the results more fragile. Future research should prioritize multicenter studies with more homogeneous groups and detailed reports of prosthetic prescriptions to reduce these confounding factors.

## 5 Conclusion

This systematic review demonstrated that individuals with amputation exhibit a functionally compromised gait, characterized by reduced walking speed and increased asymmetry. Increased step width and reduced stride length may represent a compensatory strategies to enlarge the base of support and maintain dynamic balance, thus compensating for the limited prosthetic propulsion and gait insecurity. Also, TFA generally results in greater gait impairments when compared with physically able individuals. The findings of this systematic review underscore that the influence of prosthetic component technology on gait rehabilitation still lacks scientific robustness. Above all, there is a clear need for primary studies that independently evaluate transtibial and transfemoral amputee populations. Understanding space temporal parameter alterations and functional loss stratified by amputation level is a fundamental step toward guiding both technological development and evidence‐based clinical practice.

## 6 Implications of Physiotherapy Practice

This review has a direct clinical impact on guiding gait assessment in amputee patients, particularly regarding gait speed and step width, as it presents more significant results. With the results of this review, physiotherapists can obtain information about the variation in the results of spatiotemporal parameter analysis between amputee patients and physically able individuals.

## Funding

This study was supported in part by the Coordenação de Aperfeiçoamento de Pessoal de Nível Superior (CAPES), Brazil. Luis Augusto Mendes Fontes is a recipient of a doctoral scholarship from CAPES.

## Ethics Statement

The authors have nothing to report.

## Consent

The authors have nothing to report.

## Conflicts of Interest

The authors declare no conflicts of interest.

## Data Availability

Research data are not shared.
